# Editorial: Transcriptome & metabolic profiling: an insight into the abiotic stress response crosstalk in plants

**DOI:** 10.3389/fpls.2024.1370817

**Published:** 2024-02-13

**Authors:** Poonam Yadav, Guanlin Li, Jose M. Mulet

**Affiliations:** ^1^ Institute of Environment and Sustainable Development, Banaras Hindu University, Uttar Pradesh, Varanasi, India; ^2^ School of Emergency Management, School of Environment and Safety Engineering, Jiangsu Province Engineering Research Center of Green Technology and Contingency Management for Emerging Pollutants, Jiangsu University, Zhenjiang, China; ^3^ Instituto de Biología Molecular y Celular de Plantas, Universitat Politècnica de València-Consejo superior de investigaciones científicas (CSIC), Valencia, Spain

**Keywords:** salt stress, drought stress, crops, transcriptome, metabolome

Global warming is menacing natural ecosystems (Taïbi et al., 2015) and food production systems (Melino and Tester, 2023). Abiotic stress usually affects the normal plant developmental program at several points. Drought and salt stress, i.e., less water than is required and a sodium concentration in soil of approximately 20-30 mM (glycophytes) or 100-200 mM (halophytes), induce abscisic acid, which arrests plant development (Chevilly et al., 2021). In addition, salt stress in the soil increases osmotic potential, makes water uptake more difficult for plants, and competes with the uptake of potassium, the major mineral nutrient (Yadav and Jaiswal, 2021; Mulet et al., 2023). Inside the plant, sodium is toxic and interferes with many biochemical processes, and plants must divert energy from the developmental program to maintain correct ion homeostasis (Flowers and Colmer, 2015). Similar constraints for the developmental program occur with temperature stress: freezing (below 0°C), chilling (usually between 0°C to 5°C), or heating (usually above 30°C, but depends on the plant). Heat stress also increases photorespiration and thus increases oxidation (Busch, 2020). Whereas under heavy metal toxicity overproduction of reactive oxygen species (ROS) and redox imbalance alters the energetic ratio of ATD/ADP, NADP/NADPH and NAD/NADH. Plant's compromised energetic balance are suggested as major factors related to heavy metal stress (Srivastava et al., 2013; Yadav et al., 2021). It becomes clear that the study of abiotic stress in plants cannot be carried out as a single effect but as a whole, and the crosstalk between the different signal transduction responses must be considered to obtain a general picture (Yadav et al., 2021). Systems biology, specifically technological advances in bioinformatics, and mass spectrometry, among other techniques, have allowed us to study the impact of abiotic stress on the whole transcriptome or metabolome, and thus investigate how different pathways are affected by the applied stress.

In this Research Topic, we collected twelve excellent contributions that bring new light to this complex subject and emphasize the significance of the transcriptomic and metabolomic profile in solving the complex puzzle of stress management and mechanisms in plants. Additionally, this may help the biotechnologist involved in breeding or genetic engineering to better understand the stress management mechanism and complex role of the metabolic mechanism and develop stress and climate-resilient crops in the near future.

## Abiotic stress in cereal crops

One of the advantages of recent technological advances is that abiotic stress can be studied at the molecular level not only in model organisms such as *Arabidopsis thaliana* but also in wild or cultivated plants. There is no study investigating model plants in this Research Topic but there are two that focus on barley and one that features maize. Mahalingam et al. take an original approach by comparing the transcriptome of stress-tolerant (Otis) and stress-sensitive (Golden Promise) barley genotypes subjected to drought, heat, and combined heat and drought stress for 5 days during the heading stage. The stress-sensitive phenotype presented a higher number of differentially expressed genes than the stress-resistant phenotype. Genes associated with RNA metabolism and *Hsp70* chaperones were the most upregulated; therefore, the authors suggested that these may be targets for biotechnological improvement.

It is known that during drought, plants close their stomata at a critical soil water content (SWC), and this induces diverse physiological, developmental, and biochemical responses. Paul et al. investigated this phenomenon in different genotypes of barley and found that the different responses among genotypes could suggest a specific adaptation to different rain patterns and the prominent role of the retrotransposon *BARE1*, as well as the identification of novel genes participating in the drought stress response.

Zhang et al. showed the response-related mechanism of a low phosphorus (LP)-induced gene *ZmG6PE* and its stress impact on maize (*Zea mays ssp. mays*) yield. It was advocated that the *ZmG6PE* gene was required under the LP response by mediating the expression of the *SPX6* and *PHT1.13* genes in maize plants. Additionally, the *ZmG6PE* gene contributed to the grain yield of maize through sugar and starch synthesis under LP stress. A combined transcriptomic and metabolomic study also revealed that the plant’s immune regulation was activated in response to the LP stress under the influence of carbon metabolism, fatty acid metabolism, and amino acid metabolism.

## Abiotic stress in horticultural crops

Vegetables are essential for a healthy diet. Horticultural crops are prone to abiotic stress due to their high water requirement. In this Research Topic, two studies have contributed with advances in onion (*Allium cepa*). Onion is another important global vegetable/spice crop and faces various challenges in the field. Gedam et al., investigated the impact of waterlogging (hypoxia) stress on onion. The study included in this Research Topic describes the transcriptomic response in leaves of two contrasting genotypes of onion under waterlogging stress and reports that several key biological processes were affected, such as phytohormone production, antioxidant enzymes, programmed cell death, and aerenchyma development. Additionally, changes were observed in the regulation of energy production under the stress response. Antioxidant enzyme activity was also higher in the tolerant variety than in the sensitive W-344 variety. Furthermore, they reported that some genes related to waterlogging tolerance, such as *RAP2-12* and *RAP2-3*, were highly expressed in the tolerant variety. Manape et al. identified and validated the probable genes governing wax accumulation in onion through transcriptomic profiling of the glossy mutant and its wild-type counterpart. The study revealed significant differences in the genes involved in the wax biosynthesis pathway in two different types of onions, the glossy mutant and the wild type. The study represents a significant contribution to onion resistance breeding against stress imposed by thrips (pest attack) or drought.

## Contributions to woody plants

In this Research Topic, we have also gained some insight into the abiotic stress response in woody plants. We present a report on grapevine, a woody crop of major importance, and an industrial crop, *Hevea brasiliensis*. Drought is seen as a common stress for grape cultivation. Yang et al. quantified the effect of drought stress on ‘Shine Muscat’ grapevine, presented the related consequences, and provided insights into the new growth regulator 5-aminolevulinic acid (ALA) in stress mitigation. The study reported a decline in MDA production, glutathione, ascorbic acid, and betaine along with the activation of POD and SOD to better manage stress under the application of ALA. Reduction in abscisic acid by upregulating *CYP707A1* gene helped in relieving the closure of stomata and also induced changes in some chlorophyll synthesis genes. This finding can pave the way for better drought stress management of grapes and other crops in the future.

Mao et al. explored new insights regarding the metabolomic and transcriptomic profiling of rubber plants (*Hevea brasiliensis*) under cold stress. The study presented a detailed analysis of transcriptomics and metabolomics that revealed the role of cold-stress-responsive genes and metabolite molecules in rubber trees. The finding was promising as two rubber tree clones, temperature-sensitive and cold-resistant, were studied, and their transcriptomic and metabolomic responses through RNA-seq and LCMS-based metabolite profiling were mapped. Additionally, they highlighted key pathways, such as flavonoid, arginine, and anthocyanin metabolism involved with cold resistance. It is very important to track the modulation of gene expression due to cold stress and its effect on the metabolome. Finally, we also included a study on the Chinese tree *qing qian liu*, or wheel wingnut (*Cyclocarya paliurus*). Zhang et al. investigated and demonstrated the role of two stress signaling molecules, nitric oxide (NO) and hydrogen sulfide (H_2_S), in salt stress tolerance in *Cyclocarya paliurus*. They described the intricate role of exogenous application of NO and H_2_S in salt tolerance in *C. paliurus*. The mechanisms is based in maintaining the photosynthetic ability and energetics, as evidenced by reduced leaf biomass loss. The study also reports increased cellular NO synthesis and decreased oxidative damage through the activation of the antioxidant enzymatic machinery and by increasing the soluble protein and flavonoid content.

## Abiotic stress and medicinal herbs

Medicinal plants are of major economic and social interest. Metabolomic and transcriptomic profiling is a very useful technology for identifying active ingredients and investigating how environmental conditions affect their accumulation. Zhou et al. addressed a specific problem. *Gynostemma pentaphyllum*, also called Southern Ginseng, Miracle Plant, or Jiaogulan, is an important medicinal herb but can absorb high amounts of cadmium (Cd), which may be deleterious for consumers. The study included in this Research Topic investigated the genomic and metabolomic response of this plant to cadmium stress to develop a novel cultivar that accumulates cadmium less. The authors found that phenylpropanoid biosynthesis, starch, sucrose metabolism, alpha-linolenic acid metabolism, and the ABC transporter were significantly enriched at the gene and metabolic levels.

*Illicium difengpi* is an endangered medicinal plant, native to the karst mountains of the Guangxi region of China. This plant is highly adapted to drought stress. Zhang et al. performed transcriptomic and metabolomic profiling to obtain new insights into its drought tolerance mechanism. The joint transcriptome and metabolome analyses showed that under drought there was an increase in glutathione, flavonoids, polyamines, soluble sugars, and amino acids, contributing to cell osmotic potential and antioxidant activity.

The *Pueraria* genus includes more than 20 plants species, which are economically important food and medicinal plants in South-East Asia. Fu et al. analyzed the flavonoids, dietary fiber, total starch, and crude protein of one *P. lobatae* and three *P. thomsonii* varieties by combining various chemical analysis methods. Based on their findings, they proposed *P. lobata* is better for medicinal use, whereas *P. thomsonii* is a better option as edible food.

And finally, we also contribute with a review on zinc fingers. Moulick et al. put good effort into collecting literature about the detailed molecular involvement of Zn-finger motifs in abiotic stress management in crop plants. They comprehensively reviewed Zn-finger motifs and provided deep insights into the role of Zn fingers in various abiotic stress tolerance mechanisms. The review covers structural to functional aspects of zinc finger motifs/proteins and their involvement in various signaling transduction pathways, triggering the action of plant transcription factors and controlling the expression of various stress-regulated genes.

## Outlook

It becomes crystal-clear that abiotic stress cannot be studied as an isolated process but must be considered as the different effects that are exerted on the essential processes of plant physiology and the multiple read-outs. Metabolomic and transcriptional studies have allowed us to study this process in general and unveil how different signal transduction pathways, many previously unrelated to the studied stress, are affected. These studies provide novel and valuable information on the crosstalk of different abiotic stresses and their interplay with developmental processes in crops and medicinal and woody plants. We hope that future investigations will convert this information into improved crops with increased tolerance to abiotic stress or increased nutritional content.

## Author contributions

PY: Writing – original draft, Writing – review & editing. GL: Writing – original draft, Writing – review & editing. JM: Writing – original draft, Writing – review & editing.
